# Combination Therapy and Single-Modality Treatment for Acute Low-Tone Hearing Loss: A Meta-Analysis with Trial Sequential Analysis

**DOI:** 10.3390/brainsci12070866

**Published:** 2022-06-30

**Authors:** Jing-Li Leong, Chih-Hao Chen, Chii-Yuan Huang, Hsiu-Lien Cheng, Yuan-Chia Chu, Chun-Yu Chang, Yen-Fu Cheng

**Affiliations:** 1 Department of Medical Education, Taipei Veterans General Hospital, Taipei 112, Taiwan; leongjingli@gmail.com; 2 Department of Otolaryngology-Head and Neck Surgery, Taipei Veterans General Hospital, Taipei 112, Taiwan; michaelchen808@gmail.com (C.-H.C.); dopod0635@gmail.com (C.-Y.H.); hlcheng@vghtpe.gov.tw (H.-L.C.); 3 Faculty of Medicine, National Yang Ming Chiao Tung University, Taipei 112, Taiwan; 4 Department of Biomedical Engineering, National Yang Ming Chiao Tung University, Taipei 112, Taiwan; 5 Information Management Office, Taipei Veterans General Hospital, Taipei 112, Taiwan; xd.yuanchia@gmail.com; 6 Medical AI Development Center, Taipei Veterans General Hospital, Taipei 112, Taiwan; 7 Department of Information Management, National Taipei University of Nursing and Health, Taipei 112, Taiwan; 8 Department of Anesthesiology, Taipei Tzu Chi Hospital, Buddhist Tzu Chi Medical Foundation, New Taipei City 231, Taiwan; paulchang1231@gmail.com; 9 Department of Medical Research, Taipei Veterans General Hospital, Taipei 112, Taiwan; 10 Institute of Brain Science, National Yang Ming Chiao Tung University, Taipei 112, Taiwan

**Keywords:** acute low-tone hearing loss, diuretic, steroid, combination therapy

## Abstract

Acute low-tone hearing loss (ALHL) is a common clinical disease and was first proposed by Abe in 1981 as sensorineural hearing loss confined to low frequencies. The best strategy for initiating medication is still unclear, as the superiority of steroids and diuretics is still debated, and combination therapy might yield additional benefits. However, no study regarding combination therapy has been published. The objective of this study was to evaluate the efficacy of steroid therapy versus combination therapy of diuretics with steroids by conducting a systematic review with a meta-analysis and trial sequential analysis (TSA). Studies enrolling patients with a diagnosis of acute low-tone hearing loss were considered eligible. After searching the PubMed, Cochrane Library, Embase, Scopus and Web of Science databases from inception to 31 December 2021, five studies including 433 patients were enrolled. Overall, the comparison between combination therapy with steroids and diuretics and single-modality treatment with steroids (OR, 1.15; 95% CI, 0.51 to 2.59; *p* = 0.74; *I*^2^ = 34%) and the comparison between combination therapy and treatment with diuretics alone (OR, 1.73; 95% CI, 0.93 to 3.23; *p* = 0.09; *I*^2^ = 5%) showed that combination therapy did not confer significant benefits when compared to single-modality treatments. A trial sequential analysis (TSA) showed conclusive nonsignificant results of the comparison between the combination of steroids and diuretics and a single-modality treatment. In conclusion, we reported that the combination of steroids and diuretics did not yield significant benefits when compared to single-modality treatment with steroids or diuretics. We suggest that treatment should be initiated with steroids or diuretics alone to avoid potential adverse effects.

## 1. Introduction

On the spectrum of hearing loss, acute low-tone hearing loss (ALHL) is a unique entity. ALHL was first distinguished from idiopathic sudden sensorineural hearing loss (SSHL) by Abe in 1981 as a type of sensorineural hearing loss confined to low frequencies [1]. The specific hearing impairment experienced among people with ALHL is mainly restricted to the lower frequencies of 125, 250 and 500 Hz, while normal hearing is essentially maintained at higher frequencies of 2, 4 and 8 kHz without vertigo or structural damage [2]. Most patients present with aural fullness, low-pitched tinnitus or dizziness (not true vertigo), and ALHL has been reported to have a better short-term prognosis than sudden sensorineural hearing loss (SSHL) [1]. It is more common in females in their 40s [3]. The incidence of definite ALHL has been reported to be 42.8–65.8 per 100,000 people [4,5]. The natural course of ALHL is still unclear. ALHL has been reported to have complete or partial spontaneous recovery within 3 months in the majority of patients, but the recurrence of low-tone loss or progression to Meniere’s disease (MD) may occur in 10–20% of patients. [6] The etiologies of ALHL include both autoimmunological mechanisms and endolymphatic hydrops occurring during the early stage of Meniere’s disease (MD).

Current single-modality treatment options are based on the etiology of autoimmune mechanisms and include steroids (to counteract the imbalance in Th1/Th2 lymphocytes) [7] and endolymphatic hydrops, such as diuretics (to correct the endolymphatic ion balance) [8]. Several studies have proposed that combined treatment with steroids and diuretics is beneficial for ALHL [9,10,11]. However, there is still a lack of a standardized protocol. Initial management is important because the outcome after the initial treatment of ALHL is correlated with long-term results [12]. Therefore, we aimed to explore whether combining the two treatments would provide more benefits than single-modality treatment. If the results are nonsignificant, we would be able to avoid the unnecessary use of steroids or diuretics and instead reserve these medications for salvage cases only.

A previous study compared the effectiveness of steroids and diuretics for the treatment of ALHL and found no significant difference in the recovery rate of patients [13]. The current meta-analysis compared the efficacy of single-modality treatment (steroids or diuretics alone) and combination treatment (steroids and diuretics) among patients with ALHL and further validated the results using TSA.

## 2. Materials and Methods

### 2.1. Study Design

The study was performed by a systematic review with meta-analysis that complied with the Preferred Reporting Items for Systematic Reviews and Meta-Analyses (PRISMA) guidelines [14].

### 2.2. Search Strategy

From inception through 31 December 2021, five databases, PubMed, Cochrane Library, Embase, Scopus and Web of Science, were included in the preliminary search. Four citation subsets were used under the framework of medical subject headings (MeSH) and text words: one included study on the concept with acute onset ("acute", "quick"), one included concept with low tone ("low tone" OR "low frequency"), one included disease with hearing loss ("hearing loss" OR "hearing impairment") and the last included the intentional treatment option ("steroid" OR "diuretics" OR "combination"). The full search strategy is listed in Table S1 in the supplementary material.

### 2.3. Eligibility Criteria

After screening the titles, abstracts and keywords of the identified records. Two coauthors (J.-L. Leong and C.-H. Chen) sequentially reviewed the full texts of favorable records, and the data-of-interest were extracted if they complied with the following criteria: (a) the study included patients with a diagnosis of definite acute low tone hearing loss (ALHL); (b) the study allocated patients into management with a single medication modality (e.g., steroid or diuretics) or combination therapy with steroids and diuretics, in which the medication should be given systemically, and (c) the study yielded sufficient information and an outcome of interest (e.g., the recovery rate of patients with ALHL after treatment to estimate the effect size for meta-analysis). When there was inconsistency regarding the inclusion of a study or extraction of data, a third author (Y.-F. Chang) joined meetings with the project team and provided consensus or discussion.

### 2.4. Risk of Bias Assessment

We used the Risk of Bias in Nonrandomized Studies of Interventions (ROBINS-I) tool to evaluate the methodological quality of the included studies [15], and disagreements were resolved by the third responsible author (Y.-F. Chang) by joining the project meeting and providing their consensus.

### 2.5. Statistical Analysis

We fitted the meta-analysis with a random-effects model to calculate the effect size, and the Cochran Q test and the *I*^2^ statistic were used to evaluate the statistical heterogeneity. *I*^2^ values of <50%, 50–74%,and ≥75% represent low, moderate and high heterogeneity, respectively [16]. The influence analysis of the comparison between a single modality and combination therapy was conducted with the combined effect estimates by ignoring the enrolled study sequentially. In addition, whether there were type I or type II errors due to insufficient data or power was evaluated by a trial sequential analysis (TSA). The conventional border of significance in the TSA analysis was set from −1.96 to 1.96 under an alpha value of 0.05 and a power of 80%, and the sequential monitoring boundary varied in accordance with the analysis. [17,18] The Metaphor package of R language under the operation with R studio was used in all of the statistical calculations of the meta-analysis [19], and TSA software version 0.9.5.10 Beta was operated to calculate the TSA [17,18].

## 3. Results

### 3.1. Study Identification and Selection

**Figure 1:**
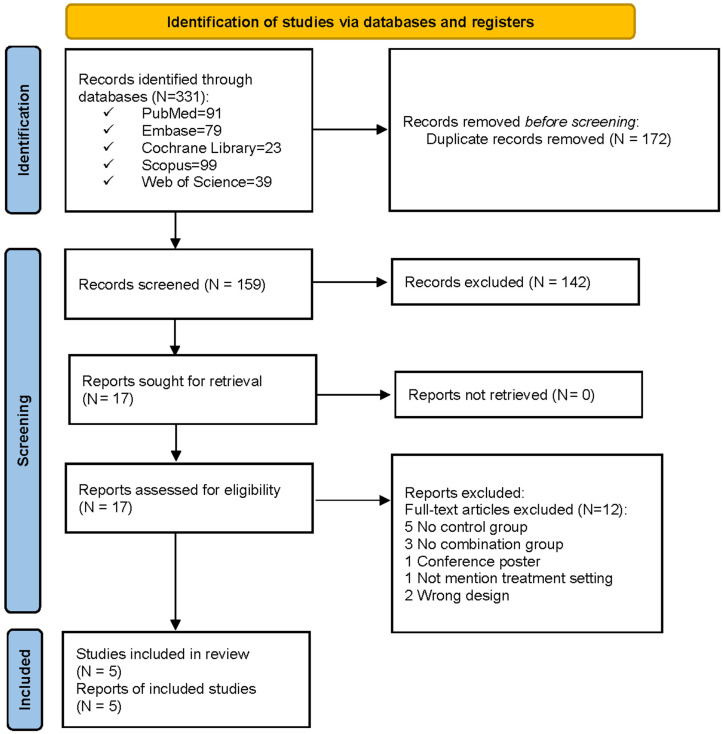
PRISMA flow diagram.

An initial search yielded 331 records. After excluding duplicates and removing records by irrelevant titles and abstracts, 17 studies underwent the full-text review, 12 of which were removed for the following reasons: no control group (*N* = 5), no combination group (*N* = 3), conference poster (*N* = 1), treatment setting not mentioned (*N* = 1), or ineligible study design (*N* = 2). Therefore, five studies were enrolled with eligibility (Figure 1).

### 3.2. Study Characteristics and Risk of Bias Assessment

A total of 433 patients from the five included studies either received treatment with steroids or diuretics alone or received combination treatment. All of the studies were composed of patients with acute low-tone hearing loss (ALHL). Four of the included studies used steroids alone for the steroid group [9,10,11,20]. Two studies were conducted in Japan [9,10], while the other three studies originated from Korea [11,20,21]. Three studies evaluated the outcome of therapy by recovery with the definition of an average hearing threshold of lower than three frequencies at 125, 250 and 500 Hz [9,10,21], while another two studies defined treatment outcomes as improvements of the average with a sum of 250 and 500 Hz [11,20]. Complete information is listed in Table 1. The risk of bias was evaluated for each of the included studies. One study was categorized as having a moderate risk of bias [20], while another study had a potentially serious risk of bias [11]. Four studies had a moderate risk of bias regarding confounding [9,10,20,21], while the study had a serious risk of bias regarding confounding [11]. One study had a moderate risk of bias regarding the classification of interventions [20]. Overall, three studies had a moderate risk of bias [9,10,21], while another study had a potentially serious risk of bias [11]. The detailed risk of bias assessment is presented in the Supplementary Section (Figures S1 and S2).

### 3.3. Outcomes

#### 3.3.1. Comparison of the Recovery Rate between Combination Therapy and Steroids Alone

**Figure 2:**
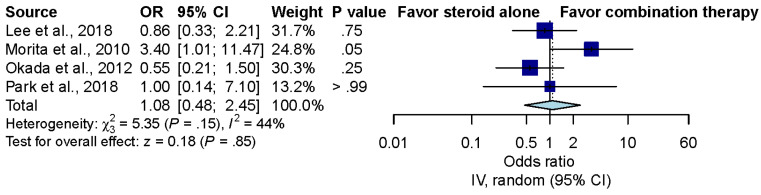
Overall effect of steroids alone and combination therapy in ALHL patients [9,10,11,20].

Four studies compared the recovery rate of ALHL patients treated with steroid treatment alone versus combination treatment with steroids and diuretics [9,10,11,20]. Overall, the pooled effect estimates showed that there was no significant difference between the steroids alone group and the combination treatment group (OR, 1.15; 95% CI, 0.51 to 2.59; *p* = 0.74; *I*^2^ = 34%) (Figure 2).

#### 3.3.2. Comparison between Combination Therapy and Diuretics Alone

**Figure 3:**
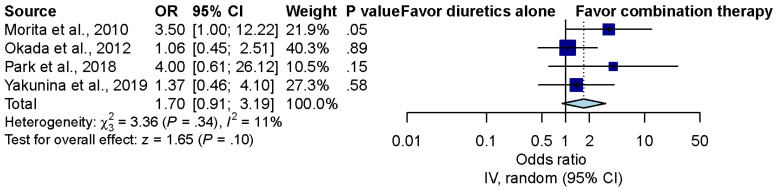
Overall effect of diuretics and combination therapy in ALHL patients [9,10,11,21].

Four studies compared the recovery rate of patients treated with diuretics alone versus combination treatment with steroids and diuretics [9,10,11,21]. Overall, the pooled results demonstrated no significant difference between the diuretics alone group and the combination treatment group (OR, 1.73; 95% CI, 0.93 to 3.23; *p* = 0.09; *I*^2^ = 5%) (Figure 3).

### 3.4. Influence Analysis

**Figure 4:**
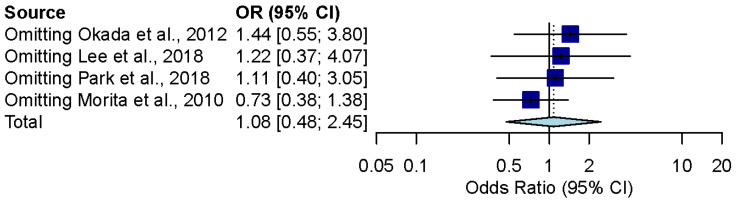
Influence analysis for the overall effect of steroid and combination therapy in ALHL patients [9,10,11,20].

**Figure 5:**
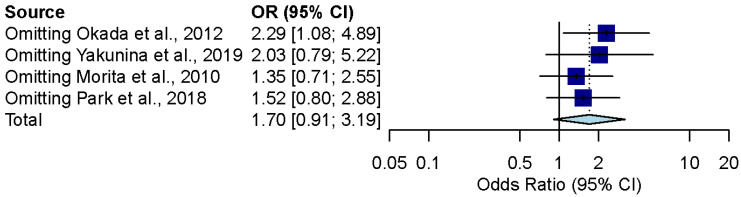
Influence analysis for the overall effect of diuretics alone and combination therapy in ALHL patients [9,10,11,21].

The combined effect estimates after removing every study one by one remained in the confidence interval of the overall results for outcomes of previous meta-analyses (Figure 4 and Figure 5).

### 3.5. Trial Sequential Analysis

**Figure 6:**
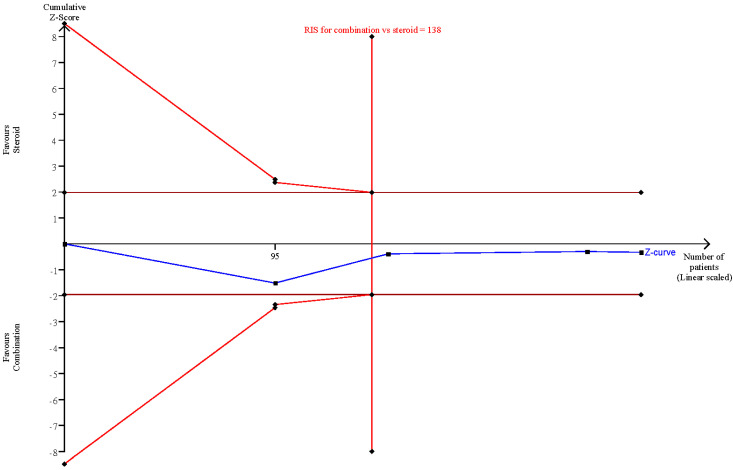
TSA comparing steroids alone and combination therapy.

**Figure 7:**
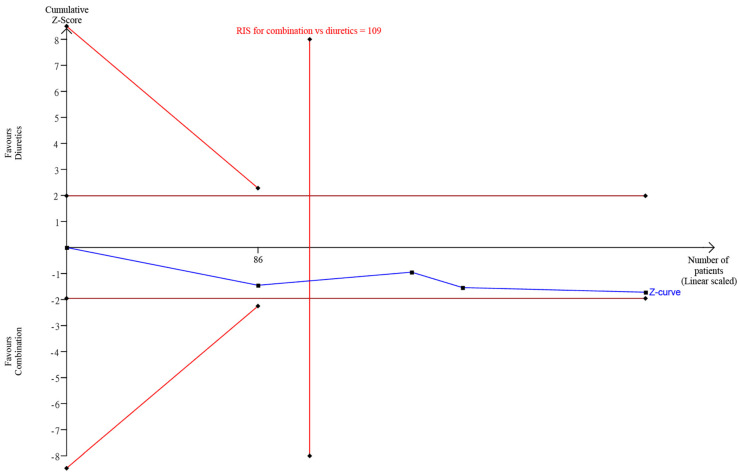
TSA comparing diuretics-alone therapy and combination therapy.

The trial sequential analysis (TSA) revealed a nonsignificant difference when comparing combination therapy and steroids-alone therapy or diuretics-alone treatment, since the RIS was reached, and the Z-curve did not surpass both the traditional significance boundary and sequential monitoring boundary (Figure 6 and Figure 7). Consequently, the TSA confirmed the abovementioned nonsignificant results.

## 4. Discussion

The primary results of the present study revealed that the combination of steroids and diuretics did not yield a significant benefit when compared to single-modality treatment with steroids or diuretics. Furthermore, the trial sequential analysis (TSA) confirmed the nonsignificant results of the comparison between the combination and single-modality treatments. Of note, all the included studies were from Japan and Korea, which may reflect the unique regional predisposition to acute low-tone hearing loss (ALHL).

At present, ALHL treatments often include steroids and diuretics. Previous studies have pointed out that there is no consensus regarding which treatment is more effective for ALHL [10,13,22]. A prior systematic review and meta-analysis of 3 RCTs assessed outcomes of hearing recovery by comparing the effectiveness of steroids and diuretics for the treatment of ALHL; the results did not show a significant difference between the recovery rate of patients treated with steroids and those treated with diuretics [13]. Given that the trials included in the current article enrolled patients undergoing treatment with both steroids and diuretics, there may be some inconsistency in evaluating the efficacy of single-modality treatment. In addition, for the meta-analysis, if the number of included articles is small, TSA is recommended to correct the results to avoid the occurrence of type I or type II errors [18]. Nevertheless, the current article provides integrated evidence for the comparison of mainstream steroid and diuretic treatments. Based on the findings that the two single-modality treatments demonstrated similar efficacy for the treatment of ALHL, we aimed to explore whether combining the two treatments together would provide more benefits than single-modality treatment. Therefore, we performed a meta-analysis of studies comparing the combination of steroids and diuretics with treatment via steroids or diuretics alone and validated the results with TSA. To our knowledge, this is the first systematic review to compare the effectiveness of single-modality treatment and combination treatment with steroids and diuretics for the management of ALHL using meta-analysis with TSA.

Currently, the pathogenesis of ALHL is not fully understood. The etiology of ALHL can be attributed to both autoimmunological mechanisms and endolymphatic hydrops. Studies have shown abnormalities in the balance of Th1/Th2 lymphocytes [7,23] in ALHL patients. This results in fluctuating inflammation in the inner ear, thus yielding inflammatory mediators (e.g., interleukin (IL)-1β and tumor necrosis factor-α), which cause inner ear tissue damage, which potentially targets the reticular lamina, stria vascularis and endolymphatic sac. Damage to the endolymphatic sac could cause an increase in permeability and sodium–potassium imbalance by increased strial sodium transport, which restores proper endolymph ion balances [8] and leads to the accumulation of water in the inner ear, further resulting in endolymphatic hydrops [24]. Electrocochleographic studies and imaging studies have identified an association between endolymphatic hydrops and ALHL [24]. Controversy regarding ALHL as the same disease spectrum of Meniere’s disease (MD) has been raised, since patients with MD also develop endolymphatic hydrops. However, only a few patients with ALHL developed classic MD with typical symptoms, including vertigo and tinnitus, after long-term follow-up, which suggested that both diseases were essentially different spectra with similar findings of endolymphatic hydrops. Recent evidence suggests that the hydrops of ALHL are confined mainly to the cochlea and would not lead to vestibular symptoms, as classic MD does [25]. Nevertheless, immunological factors and endolymphatic hydrops contribute to ALHL [24]. Further controversy exists with respect to the treatment strategies for ALHL based on whether the disorder results from the consequence of immunological factors or endolymphatic hydrops. Currently, the mainstream first-line medication for ALHL is steroids, which help to counteract this imbalance in immunological disorders. Additionally, diuretics have been reported to increase sodium transport in patients with endolymphatic hydrops [24] and effectively reduce symptoms. However, neither of the treatments showed superiority [9,13,26]. Studies have also proposed that it may be beneficial to administer both steroids and diuretics together to take advantage of their interaction, and aggressive combination treatment using steroids and diuretics should be considered for the treatment of ALHL [9,27].

Our meta-analysis suggested that a combined diuretic and steroid treatment improves hearing thresholds to a similar extent as a steroid-alone treatment, which supports the therapeutic effects of diuretics for ALHL treatment. While the result is surprising because combination therapy should directly cover both immunological and hydrops origin of ALHL, there are some possible reasons for the results. First, as mentioned in the previous paragraph, ALHL is a disorder that involves immunological factors, inflammation and eventually endolymphatic hydrops, and the process should be regarded as dynamic. Under these circumstances, we are not able to determine whether a patient’s disease severity is so mild that single-modality treatment is sufficiently effective and that combination therapy therefore does not confer additional benefits or whether the patient’s disease has progressed so severely that neither single-modality treatment nor combination therapy can effectively treat the patient. It has been pointed out that the efficacy of steroids and diuretics on ALHL is affected by the interval between the onset of symptoms and the start of treatment. According to an earlier study, when the patient started treatment within seven days after the onset of ALHL symptoms, the hearing recovery rate increased significantly [26]. The best way to investigate whether disease duration would affect the efficacy is to perform a meta-regression using time as the modulator. Unfortunately, a meta-regression only provides sufficient power when there are more than 10 included studies [28]. As a result, a. meta-regression was not suitable for the present study, and we suggest that the onset duration of disease should be clearly and appropriately described in future research. Additionally, a previous study proposed that there may be different mechanisms involved in the disease in addition to immunological factors and endolymphatic hydrops. Nozawa et al. [29]. used an orthostatic test to find that the majority of patients with ALHL have underlying autonomic nervous system disorders. Such a dysfunction may lead to transient or reversible circulatory disturbance in cochlear nerve function, which results in hearing disturbance. The situation becomes more complicated when the possible etiology involves an autonomic nervous system disorder. Various comorbidities affect the autonomic nervous system, including metabolic diseases, such as diabetes mellitus; dyslipidemia; cerebrovascular diseases and even psychiatric diseases [30,31,32,33,34]. Such nonsignificant findings may imply the involvement of pathological mechanisms other than immunological disorders and endolymphatic hydrops, and possible comorbidities may have to be adjusted in future studies. Nevertheless, the current findings indicating a nonsignificant difference between single-modality treatment and combination therapy suggest that initiating treatment with a single modality is optimal and can avoid possible drug interactions and adverse effects. For patients with limited responses, salvage combination treatment may be considered.

There are several limitations noted in our study. First, some of the included studies failed to provide sufficient information on how the patients’ randomization was applied and how the treatment allocation was concealed, which may result in insufficient reports of study design features and methodology and may affect the internal validity of trials and their associated findings. Under these circumstances, we applied the Risk of Bias in Nonrandomized Studies of Interventions (ROBINS-I) tools [15] to evaluate methodological quality, and no obvious hazard for bias was detected. Second, there was heterogeneity regarding the course in which steroids were tapered, which resulted from the experience of clinicians and different policies across institutes. Third, as previous studies have yielded the potential confounding factors that may affect the outcome of ALHL, including concurrent symptoms (e.g., vertigo or aural fullness) and comorbidities (e.g., vascular disease or migraine) [35,36,37,38], analyses for possible heterogeneity were considered for the present study. However, owing to the lack of information on characteristics and the relatively small number of included studies, additional analyses, including a meta-regression and subgroup analysis for prognostic factors associated with ALHL, could not be performed. With all of the potential heterogeneity regardless of the low level I^2^ statistic [16], we adopted a random-effects model to account for possible confounding factors that would influence the pooled effect size other than the sampling error. Finally, most of the studies did not report safety information, including adverse effects, in the single-modality and combination treatment groups, as there were some common adverse effects for the interventions (e.g., gastrointestinal bleeding or electrolyte imbalance), and safety concerns may also play a key role in treatment decisions. Large-scale and well-designed controlled trials should be conducted to overcome the abovementioned limitations.

## 5. Conclusions

We reported that the combination of steroids and diuretics did not confer a significant benefit when compared to a single-modality treatment with steroids or diuretics. Treatment should start with a single modality to prevent potential adverse effects. Further studies are needed to validate the use of our proposed system for the prediction of hearing recovery outcomes for the treatment modalities. At the same time, all the included studies were from Japan and Korea, which may reflect the unique regional predisposition to acute low-tone hearing loss.

## Figures and Tables

**Table 1:** Study Characteristic.

Study	Country	Steroid Dose (Initial Dose/Day)	Diuretic Treatment (Dose/Day)	Patients	Outcome of Combination Steroid/Diuretic	Outcome of Diuretic	Outcome of Steroid	Age (Mean ± SD, Year)	Outcome Evaluation (Hz)
Morita et al., 2010 [9]	Japan	40 mg prednisolone	90 mg Isosorbide	135	42/46	30/40	37/49	48.7 ± 10.5	Recovery defined as hearing at frequencies of 125, 250 and 500 Hz
Okada et al., 2012 [10]	Japan	30 mg prednisolone	90 mg Isosorbide	85	22/35	21/34	12/16	40.1 ± 13.2	Recovery defined as hearing at frequencies of 125, 250 and 500 Hz
Lee et al., 2018 [20]	Korea	30 mg prednisolone	90 mg Isosorbide	90	36/50	NP	30/40	43.67 ± 14.43	Recovery defined as hearing at frequencies of 250 and 500 Hz
Park et al., 2018 [11]	Korea	60 mg prednisolone	25 mg Hydrochlorothiazide	31	12/16	3/7	6/8	43.33 ± 12.92	Recovery defined as hearing at frequencies of 250 and 500 Hz
Yakunina et al., 2019 [21]	Korea	0.8 mg/kg/d of oral MPD	25 mg Hydrochlorothiazide and 40 mg isosorbide	92	34/41	32/41	NP	45.47 ± 12.67	Recovery defined as hearing at frequencies of 125, 250 and 500 Hz

## Data Availability

Not applicable.

## Supplementary Materials

The following are available online at https://www.mdpi.com/article/10.3390/brainsci12070866/s1: Table S1: Detailed search strategy; Figure S1: Detail of Risk of Bias; Figure S2: Percentage of risk of bias.
