# Supplementary material for: Combination Therapy and Single-Modality Treatment for Acute Low-Tone Hearing Loss: A Meta-Analysis with Trial Sequential Analysis
Source: Brain Sci. 2022 Jun 30;12(7):866. doi: 10.3390/brainsci12070866 (PMC9313060; doi:10.3390/brainsci12070866)
Supplement: Supplementary file 1 [file brainsci-12-00866-s001.zip › brainsci-1774911-supplementary.pdf]

Supplemental Materials

Combination Therapy and Single Modality for Acute Low-Tone Hearing Loss: A Meta-analysis with Trial Sequential Analysis

Table S1. Detailed search strategy.

Database	Query
PubMed	((("Acute Disease/drug therapy"[Mesh] OR "Acute Disease/pathology"[Mesh] OR "Acute Disease/therapy"[Mesh])) OR "Acute Disease"[Mesh] OR "acute"[TIAB] OR "urgent"[TIAB] OR "quick"[TIAB] OR "acute" OR "urgent" OR "quick") AND ("low-tone"[TIAB] OR "low-frequency"[TIAB] OR "low tone"[TIAB] OR "low frequency"[TIAB] OR "low frequencies"[TIAB] OR "low-frequencies"[TIAB] OR "low-tone" OR "low-frequency" OR "low tone" OR "low frequency" OR "low frequencies" OR "low-frequencies") AND ((("Hearing Loss/analysis"[Mesh] OR "Hearing Loss/diagnosis"[Mesh])) OR "Hearing Loss"[Mesh] OR "Hearing loss"[TIAB] OR "Hypoacusis"[TIAB] OR "Hearing Impairment"[TIAB] OR "Transitory Deafness"[TIAB] OR "Transitory Hearing Loss"[TIAB] OR "Hearing loss" OR "Hypoacusis" OR "Hearing Impairment" OR "Transitory Deafness" OR "Transitory Hearing Loss")AND((((((("Therapeutics"[Mesh]) OR ("Therapeutics/drug therapy"[Mesh] OR "Therapeutics/pharmacology"[Mesh] OR "Therapeutics/therapeutic use"[Mesh] OR "Therapeutics/therapy"[Mesh])) OR "Steroids"[Mesh]) OR ("Steroids/drug therapy"[Mesh] OR "Steroids/therapeutic use"[Mesh] OR "Steroids/therapy"[Mesh])) OR "Diuretics"[Mesh]) OR ("Diuretics/drug therapy"[Mesh] OR "Diuretics/therapeutic use"[Mesh] OR "Diuretics/therapy"[Mesh])) OR "Drug Combinations"[Mesh]) OR ("Drug Combinations/pharmacology"[Mesh] OR "Drug Combinations/therapeutic use"[Mesh] OR "Drug Combinations/therapy"[Mesh]) OR "Therapeutics"[TIAB] OR "Therapeutic"[TIAB] OR "Therapy"[TIAB] OR "Therapies"[TIAB] OR "Treatment"[TIAB] OR "Treatments"[TIAB] OR "Steroid"[TIAB] OR "Catatoxic Steroids"[TIAB] OR "Diuretic"[TIAB] OR "Diuretics"[TIAB] OR "Combination"[TIAB] OR "Combinations"[TIAB] OR "Drug Combinations"[TIAB] OR "Drug Combination"[TIAB] OR "Therapeutics" OR "Therapeutic" OR "Therapy" OR "Therapies" OR "Treatment" OR "Treatments" OR "Steroid" OR "Catatoxic Steroids" OR "Diuretic" OR "Diuretics" OR "Combination" OR "Combinations" OR "Drug Combinations" OR "Drug Combination")
Embase	(acute OR quick OR urgent) AND ('low tone' OR 'low frequency'/exp OR 'low frequencies' OR 'low frequency') AND ('hearing impairment'/exp OR hypoacusis OR 'hearing loss' OR 'transitory deafness' OR 'transitory hearing loss') AND (therapeutics OR therapeutic OR 'therapy'/exp OR therapies OR 'treatment'/exp OR treatments OR 'steroid'/exp OR 'diuretic agent'/exp OR diuretics OR diuretic OR combination OR combinations OR 'drug combinations' OR 'drug combination'/exp)

Web of Science	TS=(("acute" OR "urgent" OR "quick")AND("low-tone" OR "low-frequency" OR "low tone" OR "low frequency" OR "low frequencies" OR "low-frequencies"")AND("Hearing loss" OR "Hypoacusis" OR "Hearing Impairment" OR "Transitory Deafness" OR "Transitory Hearing Loss")AND("Therapeutics" OR "Therapeutic" OR "Therapy" OR "Therapies" OR "Treatment" OR "Treatments" OR "Steroid" OR "Catatoxic Steroids" OR "Diuretic" OR "Diuretics" OR

	"Combination" OR "Combinations" OR "Drug Combinations" OR "Drug Combination"))
Scopus	(TITLE-ABS-KEY("acute" OR "urgent" OR "quick") AND TITLE-ABS-KEY("low-tone" OR "low-frequency" OR "low tone" OR "low frequency" OR "low frequencies" OR "low-frequencies") AND TITLE-ABS-KEY("Hearing loss" OR "Hypoacusis" OR "Hearing Impairment" OR "Transitory Deafness" OR "Transitory Hearing Loss") AND TITLE-ABS-KEY("Therapeutics" OR "Therapeutic" OR "Therapy" OR "Therapies" OR "Treatment" OR "Treatments" OR "Steroid" OR "Catatoxic Steroids" OR "Diuretic" OR "Diuretics" OR "Combination" OR "Combinations" OR "Drug Combinations" OR "Drug Combination"))
Cochrane Library	Search Name: 20211223_acute_low_tone_HL_Tx<br>Last Saved: 23/12/2021 16:52:17<br>Comment:<br><br>ID Search<br>#1 MeSH descriptor: [Acute Disease] explode all trees<br>#2 MeSH descriptor: [Acute Disease] explode all trees and with qualifier(s): [therapy - TH]<br>#3 acute<br>#4 urgent<br>#5 quick<br>#6 {OR #1-#5}<br>#7 low-tone<br>#8 low-frequency<br>#9 low tone<br>#10 low frequency<br>#11 low frequencies<br>#12 low-frequencies<br>#13 {OR #7-#12}<br>#14 MeSH descriptor: [Hearing Loss] explode all trees<br>#15 MeSH descriptor: [Hearing Loss] explode all trees and with qualifier(s): [diagnosis - DI]<br>#16 Hearing loss<br>#17 Hypoacusis<br>#18 Hearing Impairment<br>#19 Transitory Deafness<br>#20 Transitory Hearing Loss<br>#21 {OR #14-#20}<br>#22 MeSH descriptor: [Therapeutics] explode all trees<br>#23 MeSH descriptor: [Steroids] explode all trees<br>#24 MeSH descriptor: [Steroids] explode all trees and with qualifier(s): [therapeutic use - TU]<br>#25 MeSH descriptor: [Diuretics] explode all trees

	#26 MeSH descriptor: [Diuretics] explode all trees and with qualifier(s): [therapeutic use - TU] #27 MeSH descriptor: [Drug Combinations] explode all trees<br>#28 Therapeutics<br>#29 Therapeutic<br>#30 Therapy<br>#31 Therapies<br>#32 Treatment<br>#33 Treatments<br>#34 Steroid<br>#35 Catatoxic Steroids<br>#36 Diuretic<br>#37 Diuretics<br>#38 Combination<br>#39 Combinations<br>#40 Drug Combinations<br>#41 Drug Combination<br>#42 {OR #22-#41}<br>#43 #6 AND #13 AND #21 AND#42

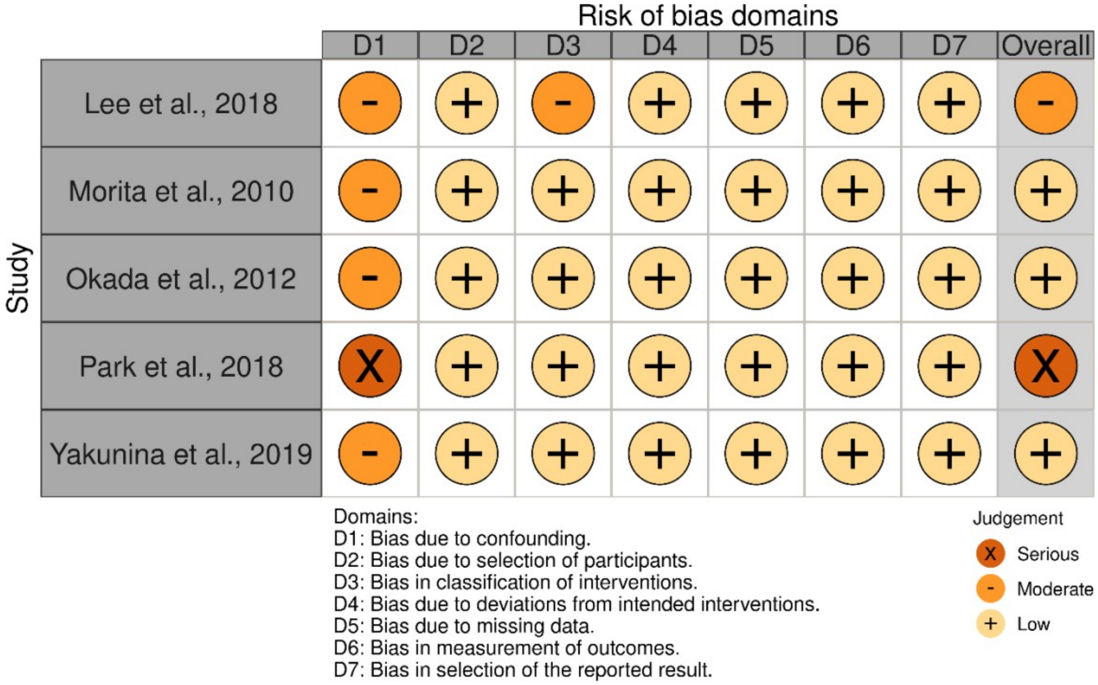

Figure S1. Risk of Bias. The Risk of Bias in Non-randomized Studies - of Interventions (ROBINS-I) was used to evaluate to quality of included studies.

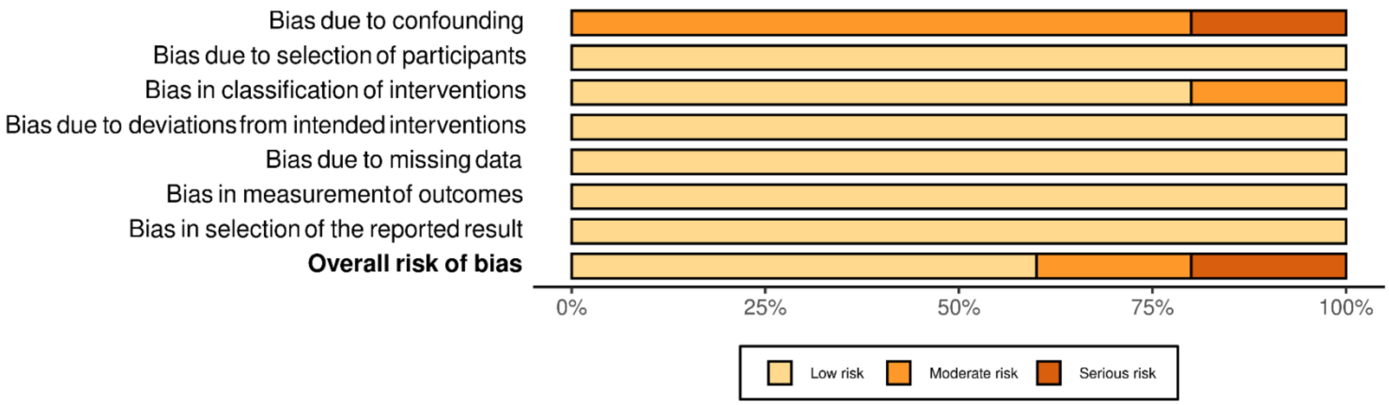

Figure S2. Summary of Risk of Bias. Overall, 20% of the studies are subject to moderate risk of bias while another 20% are subject to serious risk of bias. 80% of studies have moderate risk in bias due to confounding, while 20% of studies may sustain serious risk in bias due to confounding. 20% of studies have moderate risk in classification of interventions.
